# The role of arts engagement in reducing cognitive decline and improving quality of life in healthy older people: a systematic review

**DOI:** 10.3389/fpsyg.2023.1232357

**Published:** 2023-08-21

**Authors:** Massimo Fioranelli, Maria Grazia Roccia, Maria Luisa Garo

**Affiliations:** ^1^Department of Human Sciences, Guglielmo Marconi University, Rome, Italy; ^2^Istituto Terapie Sistemiche Integrate, Casa di Cura Sanatrix, Rome, Italy

**Keywords:** arts engagement, cognitive decline, quality of life, well-being, healthy population, elderly

## Abstract

In recent years, arts engagement has been proposed as a non-pharmacological approach to reduce cognitive decline and increase well-being and quality of life in specific populations such as the elderly or patients with severe disease. The aim of this systematic review was to assess the effects of receptive or active arts engagement on reducing cognitive decline and improving quality of life and well-being in healthy populations, with a particular focus on the role of arts engagement in the long term. A comprehensive search strategy was conducted across four databases from February to March 2023. Ten studies with a total of 7,874 participants were incorporated in accordance with the PRISMA guidelines. Active and receptive arts engagement was found to be an effective approach to reduce cognitive decline and improve well-being and quality of life in healthy populations. The role of the positive effects of arts engagement could be determined by the combination of several factors such as exposure to cultural activities and the group effect. There is limited evidence of the protective effects of active arts engagement over a long period of time. Given the increasing demand for preventive programmes to reduce the negative effects of population ageing, more research on arts engagement should be conducted to identify its mechanisms and long-term effects.

## 1. Introduction

According to a recent report by the World Health Organisation (WHO), engaging in the arts offers a wide range of health benefits, from supporting social determinants of health to preventing mental and physical illness and helping manage and treat various health conditions such as cancer, dementia, schizophrenia, anxiety and depression (Fancourt and Finn, 2019).

Engaging in art promotes the process of creativity and autonomy that cultivates mindfulness, self-knowledge and new insights and involves a number of physiological mechanisms such as stimulating the parasympathetic nervous system or neuroplasticity and building cognitive reserves, enhancing social interaction or changing lifestyle habits such as reducing sedentary behaviour (Weiss et al., 1989; Lane, 2005; Vance et al., 2012; Bolwerk et al., 2014; Poulos et al., 2019; Odeh et al., 2022). Artistic activities are indeed multimodal health interventions that combine several psychological, physical, social and behavioural factors and include a relevant aesthetic engagement (Fancourt, 2017). They offer people the opportunity to explore personal problems without relying on a verbal form of communication as well as helping them deal with symptoms, stress and traumatic experiences in their lives and to connect with their inner selves (Kim, 2013; Stevens et al., 2019; Moula et al., 2020).

Arts engagement is generally understood to have a broad definition involving artistic creativity expressed or experienced by people. Formally, arts engagement can be defined as active (e.g., creating or making art) or receptive (e.g., attending or viewing art) participation in creative events or activities within a variety of art forms (Davies et al., 2016; Davies and Clift, 2022). Active or receptive participation in visual arts, theatre, literature or music has been shown to contribute significantly in increasing the well-being and quality of life, reducing the risk of illness, accelerating disease recovery, increasing life expectancy, reducing grief and negative emotions, as well as improving immune system response, slowing disease progression, promoting positive social contact, enhancing cognitive status (Cohen et al., 2006; Kim, 2013; Schneider, 2018), and reducing depression and anxiety (Mann et al., 2017). With its potential to help individuals express themselves, gain coping skills, improve interpersonal skills, resolve conflicts and problems, reduce stress, manage behaviour, increase self-esteem and self-confidence (Davies et al., 2016; Davies and Clift, 2022; Mollaoglu and Yanmis, 2022; Shukla et al., 2022), arts engagement is proposed as a non-pharmacological therapeutic approach with significant effect in alleviating chronic stress and depression and in providing emotional, cognitive and social coping resources that support biological regulatory systems (Beerse et al., 2020). Moreover, it also has a positive impact on social capital by helping people in reducing loneliness (Gordon-Nesbitt, 2015; Roe et al., 2016).

A recent prospective longitudinal study conducted on a sample of 6,710 community-dwelling adults aged 50 and older found that arts may have a protective effect on longevity by affecting cognition, mental health and physical activity, and establishing a kind of dose–response relationship with longevity such that people who engaged in arts activities infrequently (once or twice a year) had a 14% lower risk of death than those who did not, and those who engaged frequently (every few months or more often) had a 31% lower risk (Fancourt and Steptoe, 2019).

To date, several studies conducted have shown a stronger association between arts engagement, improved cognitive function, increased well-being and quality of life in patients with certain medical diagnoses such as cancer or dementia (Jiang et al., 2020; Chacur et al., 2022; Letrondo et al., 2023). In addition, arts engagement has been shown to have a protective effect against cognitive decline, in reducing distress and discomfort, and in mitigating loneliness or hopelessness in the elderly (Chacur et al., 2022). However, the role of the arts in reducing cognitive decline and improving quality of life and well-being in healthy populations [i.e., optimal physical and mental functioning, absence of debilitating diseases, and delayed age-associated disease onset (Behr et al., 2023)] is much less studied (Fancourt and Steptoe, 2019; Galassi et al., 2022). To date, a few reviews, primarily scoping reviews, have been conducted on the role of creativity, art therapy, and group-based arts interventions in reducing cognitive decline and improving quality of life in older adults, showing that active engagement in arts activities such as dance, music, or song plays a role in promoting health and mitigating disease in older adults (Fraser et al., 2015; Galassi et al., 2022; McQuade and O'Sullivan, 2023). To our knowledge, there are no systematic reviews of the role of arts engagement in healthy populations that address the potential role of arts engagement in reducing cognitive decline and improving long-term quality of life and well-being. Hence, the aim of this systematic review was to assess the impact of receptive or active engagement with the arts on the reduction of cognitive decline and the improvement of the quality of life and well-being in healthy populations. Specifically, we aimed to answer the following two research questions:

1. Does arts engagement improve cognitive function?
2. Does arts engagement improve quality of life and well-being in the long term?

## 2. Methods

A detailed systematic review of published data was performed according to the PRISMA (Preferred Reporting Items for Systematic Reviews and Meta-Analyses) guidelines (Moher et al., 2009). The methodological approach was registered in the PROSPERO database under the protocol number CRD42023414916.

### 2.1. Eligibility criteria

We included studies published (i.e., peer-reviewed journal articles) that evaluated adult (age ≥ 18 years) healthy individuals without cancer, schizophrenia, or dementia diagnosis. No exclusions were made based on gender, ethnicity or socioeconomic status of participants.

### 2.2. Studies design

We included observational studies and randomised controlled trials (RCTs), quasi-RCTs and non-RCTs. Quasi-randomisation was defined as allocation that is not truly random but intend to produce balanced groups (e.g., allocation by date of birth or alternation). Qualitative studies were not included because of possible large heterogeneity due to different approaches, that could affect the understanding of the net role of arts engagement, especially in the case of receptive arts engagement.

We included studies that evaluated cognitive decline, quality of life, and/or well-being in the healthy population that was involved in art engagement activities, such as visual arts, dance, drama, poetry, reading, storytelling, collage, pottery, museum/gallery visits and painting, considering a variety of settings such as community centres, parks, workplaces, schools, universities, museums, theatres, art galleries, concert halls or online. Studies involving a contemporaneous evaluation of the effect of art engagement and other activities (e.g., gardening or physical activities) or which only determined art therapy impact on anxiety or self-esteem were excluded.

### 2.3. Outcome

The primary outcomes were cognitive decline parameters (results from mini-mental state examination, word-list recall, delayed word-list recall, category fluency, digit span, story recall task, problem solving); quality of life (score from Quality-of-Life questionnaire or Life Satisfaction scale) and well-being (Warwick-Edinburgh Mental Well-being Scale, Loneliness Scale or Hopelessness Scale).

### 2.4. Search strategy and study selection

A systematic search was carried out on PubMed, Web of Science, Cochrane Library, and Scopus from February to March 2023 without time and language restrictions. The literature search strategy was based on the following keywords: (“arts engagement” OR “art therapy” OR “arts intervention”) AND (cognitive function OR “cognitive decline” OR cognition OR “cognitive impairment” OR “quality of life” OR QoL OR mortality OR well-being). The first (title/abstract screening) and second (full-text assessment) steps of the search process were performed by two independent reviewers (MGR and MLG), and any disagreement was discussed until a consensual decision was made with a third experienced reviewer (MF).

The complete list of articles obtained through the systematic search was screened to remove duplicates and exclude ineligible articles. The potentially relevant articles that answer the research questions were screened by reading titles and abstracts. Two reviewers (MGR and MLG) independently selected the eligible studies. Full texts of the remaining potentially relevant articles that met the inclusion and exclusion criteria were retrieved. The final eligibility of each study was independently assessed by each reviewer using the above eligibility criteria. Studies that did not meet the eligibility criteria, whose study design was not defined, or whose reporting was incomplete were excluded. The reasons for exclusion were recorded. All authors executed the definitive article selection. When there was disagreement, it was solved by consensus with a third experienced reviewer (MF).

### 2.5. Data extraction

Two reviewers (MGR and MLG) independently extracted data from included studies and recorded them in a datasheet. In this case also, any disagreement was resolved by consensus. The data collected included (1) study characteristics (name of the first author, year, study design, aims, number of participants); (2) arts engagement intervention or activity; and (3) main outcomes and analysis methods. No numerical information was extracted from the figures reported in the study publications.

### 2.6. Risk of bias assessment

Two authors (MGR and MLG) independently assessed the risk of bias of included studies using RoB2 in case of RCT,^1^ NIH Tool for Before-After study without control group and for cross-sectional studies.^2^ The RoB2 algorithm was fully applied in the assessment of the studies, evaluating the potential bias in the context of specific study. For both NIH instruments, after evaluating the risk of bias related to each negative response, the final assessment was performed independently by the two reviewers. Disagreements were resolved by consensus with a third experienced reviewer (MF).

### 2.7. Data synthesis

Results were presented as a narrative summary in which studies characteristics were reported in detail. A deeper analysis about the possible difference in art form and active vs. receptive participation were explored in the synthesis.

## 3. Results

### 3.1. Search results

**Figure 1:**
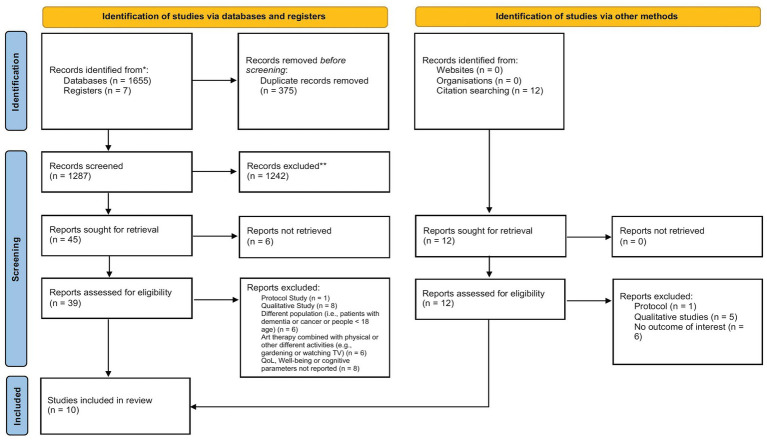
PRISMA flowchart.

The search strategy retrieved 1,662 articles from databases (PubMed: 154; Web of Science: 540; Scopus: 961; Cochrane Library: 7) (Figure 1). After excluding duplicates (*n* = 375) through EndNote (The EndNote Team, 2013), 1,287 articles were screened by reading the title and abstract. Forty-five articles met the inclusion criteria and were subsequently screened in full text. Of these, six were not found; one was excluded because it was a protocol; eight were excluded because they were qualitative studies; six included patients with dementia or cancer or patients who are <18 years; six studies combined art therapy with other activities such as gardening or physical activity and, finally, eight did not report quality of life, well-being or cognitive parameters as a quantitative outcome. Twelve further studies were found through a citation search; the reasons for exclusion are listed in Figure 1. Finally, 10 studies with a total of 7,874 participants (Noice et al., 2004; Noice and Noice, 2008; Thomson and Chatterjee, 2016; Fancourt and Steptoe, 2018; Cetinkaya et al., 2019; Ho et al., 2019; Beauchet et al., 2020; Tymoszuk et al., 2020; Aydin and Kutlu, 2021; Johnson et al., 2021) were included in the study. The average age of the patients was around 70 years, with the exception of one study that reported patients with an average age of over 80 years (Noice and Noice, 2008).

### 3.2. Characteristics of the studies

Three of the 10 studies were conducted in the USA, three in the UK, two in Turkey, one in Canada and one in Singapore. Five studies were prospective longitudinal studies, three were randomised controlled trials, one was a non-randomised clinical trial and one was a cross-sectional study. Two studies started in 2004–2005 and had an observation period of 10 years (Fancourt and Steptoe, 2018; Tymoszuk et al., 2020). The remaining studies were conducted between 2015 and 2018. Well-being and quality of life were examined in six studies, cognitive parameters were examined in four studies, while quality of life was examined in four studies along with other outcomes. Well-being and quality of life were assessed using different approaches, while cognitive parameters were analysed using similar tests: word recall task, listening span task, problem solving, delayed word-list recall, category fluency, digit span or story recall task. A complete description of included studies characteristics is reported in Table 1.

**Table 1:** Studies characteristics.

Author	Country	Study design	Period	Inclusion/exclusion criteria	Intervention	Intervention description	Interaction	Comparator	Duration	Outcome measurement
Aydin and Kutlu (2021)	Turkey	RCT	May–October 2018	Individuals over 65 years old, Minimal Mental State Examination scale ≥19, Beck Depression Scale <30, UCLA-LS ≥ 32, BHS ≥ 4, no perception of disorder that could prevent communication, no neurological or physical condition that could affect hands, no mental disorder diagnosis	Weekly clay-based art therapy sessions of 60–90 min	Clay-based art therapy	Active	Two 30-min face-to-face interviews about health, social life, and financial issue topics	6 weeks	UCLA Loneliness Scale (UCLA-LS), Beck Hopelessness Scale (BHS)
Beauchet et al. (2020)	Canada	Prospective cohort study	January–June 2018	Community-dwelling older adults over 65 years old, having Internet access where they lived, understanding and writing French or English, life expectancy estimated over 3 months, no planned relocation outside of the recruitment living area in the follow-up period of the study and concomitant participation in another experimental study	Weekly art-based activity known as “Thursday at the Museum” – 1.5 h once a week	Art-based activity in a group during which the participants were engaged directly in the creative process. Type of art activities: abstract painting, book binding, rolled paper, drawing live-model, mini-fanzine, stained glass painting	Active	NA	12 weeks	EuroQoL-5D (EQ-5D) - Warwick-Edinburgh Mental Well-being Scale (WEMWBS)
Cetinkaya et al. (2019)	Turkey	RCT	May–October 2015	Residing in the nursing home during the study period, lack of dementia, visual problem or agitation diagnosed by a physician, not being bed bound, and accepting to participate in the study voluntarily	Ceramic painting sessions (30–35 min) twice a week	Painting a ceramic object at each session	Active	No activity	8 weeks	Mini-Mental State Examination (MMSE) – Life Satisfaction Scale (LSS)
Fancourt and Steptoe (2018)	UK	Prospective longitudinal study	2004-2005	Adults aged 50 o over living in England	Culture engagement	Culture engagement: going to (1) an art gallery, museum, or exhibition; (2) theatre, a concert, or the opera; (3) the cinema	Passive	NA	10 years	Test of semantic fluency (which is often regarded as a measure of executive function since it involves self-initiated activity, organisation and abstraction, response inhibition and set shifting and calls upon memory and language processes) and an immediate and delayed recall task as a measure of memory
Ho et al. (2019)	Singapore	Cross-sectional study	2016–2017	Community dwelling Singapore residents, who were able to communicate in either English, Malay, Mandarin, Tamil, Hokkien or Cantonese. The exclusion criteria were individuals who were visibly too ill or frail to participate or were unable to provide informed consent due to cognitive impairment as assessed by a screening question during recruitment and continuous observation throughout a face-to-face survey interview.	Active art engagement (cultural activities, e.g., creating) and Passive engagement (attending, viewing or listening cultural material)	Music, dance, theatre, literary arts, visual arts, heritage activities, film and handicraft	Both	NA	NA	WHO Quality of Life Instrument (WHOQoL-8); Short Form 20 (SF-20); Functional Assessment of Chronic Illness Therapy-Spiritual (FACIT-SP-12); Support Evaluation List Short Form (ISEL-S)
Johnson et al. (2021)	USA	Mixed method approach	June–July 2018	Age 55 and over, living in one of the four affordable housing sites, having sufficient visual and hearing acuity (with assistive devices), and being fluent in English (self-report of fairly well to very well). Exclusion criteria included having a self-reported diagnosis of dementia, a serious medical condition that would interfere with participation in the study, or plans to move out of the area within 6 months.	90 min one a week	Visual art: ceramics intervention; Literature art: creative writing intervention	Active	No activity	12 weeks	Interest in life (National Institutes of Health Toolbox Apathy Scale), Personal growth and Perceived mastery (Psychosocial and Lifestyle Questionnaire), Loneliness (NIH Toolbox Loneliness Scale), General belongingness (General Belongingness Scale), Perceived neighbourhood cohesion (Self-perception of neighbourhood social cohesion)
Noice et al. (2004)	USA	No-RCT	NR	The participants were all in reasonably good health as defined by our criteria (having good vision and hearing, being able to move about the room freely, being able to drive themselves to the training site, and having no health problems that, barring an unforeseen emergency, could prevent their attendance at every session for the entire course).	Nine 90-min sessions	The theater course (acting); Visual arts course: speculating on the intention of the artist	Active	No art activity	4 weeks	Word recall task, Listening span task, Problem solving, Self-esteem scale, Psychological Well-Being Scale
Noice and Noice (2008)	USA	RCT	NR	Being over 65 years of age, possessing sufficiently good vision (with or without glasses) to read instructional materials that were printed in very large bold type (20 pt.), and being able to move about the training area (although approximately half of the participants used canes, walkers, wheelchairs, or motorised chairs)	Twice-weekly, 1-h classes	Multi-modal approach: mental-physical-emotional exercises similar to those given to beginning acting students in college and university theatre programmes. Homework consisted of watching or listening to professional performers (actors or singers) on TV or recordings.	Active	No activity	4 weeks	Word list recall, Delayed word list recall, East Boston Memory Test, Category Fluency, Digit Span, Story recall task, Problem Solving
Thomson and Chatterjee (2016)	UK	Cohort study	NR	Older adults without diagnosis of dementia	One session of 30–40 min (one-to-one or small groups)	Museum objects comprising archaeological artefacts (amulets, flint tools, pottery), artwork (engraving plates, prints), geology samples (fossils, rocks, minerals), and zoology specimens (horns, shells, teeth) were selected from university collections on the basis of visual, tactile, and kinaesthetic properties. Facilitator-led, 30 to 40 min sessions handling and discussing museum objects	Passive	NA	Only one session	Positive Affect and Negative Affect Schedule (PANAS) and Subjective wellness ad happiness (Visual Analogue Scale)
Tymoszuk et al. (2020)	UK	Prospective longitudinal study	2004-2005 and 2014-2015	English population of people aged greater than or equal to 50 years established in 2002	Culture engagement	Art engagement measured through the number of frequency of visits to (1) cinema, (2) art galleries, exhibition or museums, (3) theatre, concerts, or the opera	Passive	NA	10 years	Pleasure domain of the Control, Autonomy, Self-realisation, Pleasure scale (CASP-19); Dieners’s life satisfaction scale; Self-realisation domain of the CASP-19 and Control/Autonomy domain of a shortened version of the CASP-19

### 3.3. Risk of bias

All three RCTs had high or moderate risk of bias in the measurement of the outcome or the selection of the reported outcome. In particular, concerns arose in all three studies regarding the investigator’s awareness of the group assignment of participants and a possible influence of this awareness in the evaluation of the received intervention. Some concerns arose in the selection of the reported outcome: the presence of a pre-specified analysis plan was not reported, and it was unclear whether there were multiple people who provided ratings. The full report can be found in Table 2.

**Table 2:** Risk of bias assigned by ROB2 for randomised controlled trials.

Authors	Experimental	Comparator	Outcome	D1	D2	D3	D4	D5	Overall
Aydin and Kutlu (2021)	Clay-based art therapy	Two 30-min face-to-face interviews about health, social life, and financial issue topics	Loneliness, hopelessness	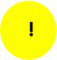	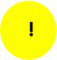	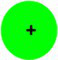	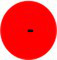	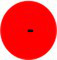	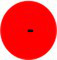
Cetinkaya et al. (2019)	Painting a ceramic object at each session	No activity	Cognitive parameters and life satisfaction	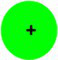	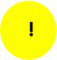	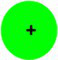	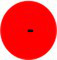	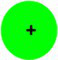	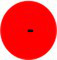
Noice and Noice (2008)	Multi-modal approach in theatre programmes.	No activity	Cognitive parameters	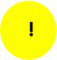	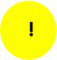	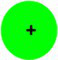	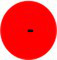	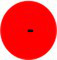	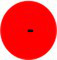

D1, Randomisation process; D2, Deviations from the intended interventions; D3, Missing outcome data; D4, Measurement of the outcome; D5, Selection of the reported results. 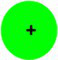, Low risk; 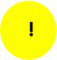, Some concerns; 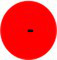, High Risk.

For observational studies, the absence of blinded assessors and the absence of multiple measurements before the intervention and in subsequent phases were reported for all included studies. In addition, in two studies, the sample size was not large enough. The risk of bias for before-after studies without a control group and for cross-sectional studies is reported in Tables 3, 4.

**Table 3:** Risk of bias assigned by NIH tool for before-after study without control group.

Authors	1	2	3	4	5	6	7	8	9	10	11	12
Beauchet et al. (2020)	YES	YES	YES	YES	YES	YES	YES	NO	YES	YES	NO	NA
Thomson and Chatterjee (2016)	YES	YES	YES	YES	NO	YES	YES	NO	YES	YES	NO	NA
Tymoszuk et al. (2020)	YES	YES	YES	YES	YES	YES	YES	NO	YES	YES	NO	NA
Fancourt and Steptoe (2018)	YES	YES	YES	YES	YES	YES	YES	NO	YES	YES	NO	NA
Johnson et al. (2021)	YES	YES	YES	YES	YES	YES	YES	NO	YES	YES	NO	NA
Noice et al. (2004)	YES	YES	YES	YES	NO	YES	YES	NO	YES	YES	NO	NA

1. Was the study question or objective clearly stated? 2. Were eligibility/selection criteria for the study population prespecified and clearly described? 3. Were the participants in the study representative of those who would be eligible for the intervention in the general or clinical population of interest? 4. Were all eligible participants that met the prespecified entry criteria enrolled? 5. Was the sample size sufficiently large to provide confidence in the findings? 6. Was the intervention clearly described and delivered consistently across the study population? 7. Were the outcome measures prespecified, clearly defined, valid, reliable, and assessed consistently across all study participants? 8. Were the people assessing the outcomes blinded to the participants’ interventions? 9. Was the loss to follow-up after baseline 20 or less? Were those lost to follow-up accounted for in the analysis? 10. Did the statistical methods examine changes in outcome measures from before to after the intervention? Were statistical tests done that provided p values for the pre-to-post changes? 11. Were outcome measures of interest taken multiple times before the intervention and multiple times after the intervention (i.e., did they use an interrupted time-series design)? 12. If the intervention was conducted at a group level (e.g., a whole hospital, a community, etc.) did the statistical analysis take into account the use of individual-level data to determine effects at the group level?

**Table 4:** Risk of bias assigned by NIH tool for cross-sectional studies.

Authors	1	2	3	4	5	6	7	8	9	10	11	12	13	14
Ho et al. (2019)	YES	YES	CD	YES	YES	CD	YES	YES	YES	NO	YES	CD	YES	YES

1. Was the research question or objective in this paper clearly stated? 2. Was the study population clearly specified and defined? 3. Was the participation rate of eligible persons at least 50? 4. Were all the subjects selected or recruited from the same or similar populations (including the same time period)? Were inclusion and exclusion criteria for being in the study prespecified and applied uniformly to all participants? 5. Was a sample size justification, power description, or variance and effect estimates provided? 6. For the analyses in this paper, were the exposure(s) of interest measured prior to the outcome(s) being measured? 7. Was the timeframe sufficient so that one could reasonably expect to see an association between exposure and outcome if it existed? 8. For exposures that can vary in amount or level, did the study examine different levels of the exposure as related to the outcome (e.g., categories of exposure, or exposure measured as continuous variable)? 9. Were the exposure measures (independent variables) clearly defined, valid, reliable, and implemented consistently across all study participants? 10. Was the exposure(s) assessed more than once over time? 11. Were the outcome measures (dependent variables) clearly defined, valid, reliable, and implemented consistently across all study participants? 12. Were the outcome assessors blinded to the exposure status of participants? 13. Was loss to follow-up after baseline 20% or less? 14. Were key potential confounding variables measured and adjusted statistically for their impact on the relationship between exposure(s) and outcome(s)?

### 3.4. Art engagement and cognitive function

Four studies (Noice et al., 2004; Noice and Noice, 2008; Fancourt and Steptoe, 2018; Cetinkaya et al., 2019) investigated the relationship between artistic activity and cognitive functions (Table 5): Three of these studies compared, in an experimental setting, the cognitive improvement of older participants who actively participated in some form of artistic activity (e.g., ceramic painting, theatre or fine arts classes) with a control group who were treated without any form of art or other leisure activities (Noice et al., 2004; Noice and Noice, 2008; Cetinkaya et al., 2019), while the fourth study examined the effects of receptive arts engagement on cognitive functions over an observation period of 10 years (Fancourt and Steptoe, 2018).

**Table 5:** Results of studies analysing the impact of arts engagement on cognitive decline.

Author	Sample	Intervention sample	Comparator sample	Male/female ratio	Age	Endpoints	Outcome	Outcome measurement	Results
Cetinkaya et al. (2019)	30	15	15	21/11	74.5 ± 9	Before, after 8 weeks	Cognitive parameters and life satisfaction	Mini-Mental State Examination (MMSE)	MMSE: IG: 24.2 ± 3.6; CG: 21.4 ± 4.3 (*p* < 0.001)
Fancourt and Steptoe (2018)	3445	NA	NA	1543/1902	62.9 ± 7.5	At enrolment (2004/2005); After 10 years (2014/2015)	Cognitive decline	Test of semantic fluency (which is often regarded as a measure of executive function since it involves self-initiated activity, organisation and abstraction, response inhibition and set shifting and calls upon memory and language processes) and an immediate and delayed recall task as a measure of memory	Going to galleries and museums A smaller decline in cognitive function compared with non-participation. For memory: a dose–response relationship indicating that more frequent attendance had a greater effect on cognition. For semantic fluency, attending once a year or more appeared to be protective Going to the theatre, concert or opera. A smaller decline in cognitive function. In relation to both memory and semantic fluency, attending once a year or more appeared to be protective with evidence of a dose–response relationship in particular for semantic fluency indicating that more frequent attendance had a greater effect on cognition. Going to the cinema. Significantly associated with cognition when taking baseline cognitive function and demographic factors into account, results became inconsistent in the fully adjusted models. For memory, attending every few months appeared to be protective, but there was no evidence that attending either more or less frequently than this had any benefits. For semantic fluency, only attending on an infrequent basis appeared to have any protective effect and these results were attenuated when correcting for multiple comparisons.
Noice et al. (2004)	124	Theatre: 44; Visual: 36	31	27/97	73.7 ± 5.99	At baseline; after 4 weeks	Cognitive parameters and well-being	Word recall task, Listening span task, Problem solving, Self-esteem scale, Psychological Well-Being Scale	Word recall Theatre: 17.16 ± 3.91, Visual: 15.83 ± 4.07; Control: 13.10 ± 4.53, *p* = 0.02; Total memory span: Theatre: 24.39 ± 2.72, Visual: 22.89 ± 5.10, Control: 21.58 ± 4.92, *p* > 0.05; Problem solving: Theatre: 8.89 ± 3.75, Visual: 4.56 ± 2.16, Control: 6.13 ± 3.33, *p* < 0.001; Self-esteem: Theatre: 3.64 ± 0.37; Visual: 3.37 ± 0.40, Control: 3.49 ± 0.36, *p* > 0.05; Psychological Well-being Theatre: 5.50 ± 0.52; Visual: 4.90 ± 0.57; Control: 4.97 ± 0.59, *p* = 0.001
Noice and Noice (2008)	122	Theatre: 42; Voice: 40	40	Theatre: 8/34; Voice: 7/33; Control: 4/36	Theatre: 80.24 ± 6.47; Voice: 82.65 ± 4.67; Control: 81.60 ± 5.96	Baseline; after 4 weeks	Cognitive parameters	Word list recall, Delayed word list recall, East Boston Memory Test, Category Fluency, Digit Span, Story recall task, Problem Solving	Digit span forward Theatre: 7.76 ± 1.89, Voice: 7.25 ± 2.04, Control: 7.23 ± 1.94; Digit span backward Theatre: 5.62 ± 1.87, Voice: 5.43 ± 1.63, Control: 5.53 ± 1.83; EBM (immediate) Theatre: 9.71 ± 1.95, Voice: 9.05 ± 1.92, Control: 8.80 ± 2.17; EBM (delayed) Theatre: 9.86 ± 1.75, Voice: 9.05 ± 1.92, Control: 8.33 ± 2.35; Problem Solving Theatre: 9.98 ± 2.68, Voice: 7.45 ± 2.55, Control: 6.78 ± 2.65, *p* < 0.001; Verbal Fluency Theatre: 37.02 ± 9.20, Voice: 29.68 ± 7.08, Control: 30.25 ± 5.88, *p* < 0.001; Word recall (immediate) Theatre: 24.31 ± 3.95, Voice: 20.48 ± 4.41; Control: 19.63 ± 3.70, *p* = 0.001; Word recall (delayed) Theatre: 7.83 ± 2.19; Voice: 6.08 ± 2.73; Control: 6.13 ± 2.12, *p* < 0.05.

In the randomised controlled trial conducted on a sample of 122 participants, Noice and Noice (2008) demonstrated that active theatre and voice activities conducted in two groups of 42 and 40 participants, respectively, over a four-week period significantly improved the problem-solving ability compared to the control group (*n* = 40) who were without any form of activity (theatre: 9.98 ± 2.68, voice: 7.45 ± 2.55, control: 6.78 ± 2.65, *p* < 0.001); verbal fluency (theatre: 37.02 ± 9.20, voice: 29.68 ± 7.08, control: 30.25 ± 5.88, *p* < 0.001); immediate word recall (theatre: 24.31 ± 3.95, voice: 20.48 ± 4.41; control: 19.63 ± 3.70, *p* = 0.001); and delayed word recall (theatre: 7.83 ± 2.19; voice: 6.08 ± 2.73; control: 6.13 ± 2.12, *p* < 0.05). For other cognitive parameters, such as digit span and the East Boston Memory Test, participants reported no significant improvement compared to the control group (Noice and Noice, 2008). In the context of a more rigorous study design, these results confirmed what the same authors had already demonstrated in an earlier study (not RCT) in 2004. In that study, conducted on a sample of 124 participants (mean age 73.7 ± 5.99 years) randomly divided into three groups (44 in the theatre group, 36 in the voice group and 31 in the control group), Noice et al. (2004) showed a significant improvement in word recall (theatre: 17.16 ± 3.91, visual: 15.83 ± 4.07; control: 13.10 ± 4.53, *p* = 0.02); total memory span (theatre: 24.39 ± 2.72, visual: 22.89 ± 5.10, control: 21.58 ± 4.92, *p* > 0.05); problem solving (theatre: 8.89 ± 3.75, visual: 4.56 ± 2.16, control: 6.13 ± 3.33, *p* < 0.001); and phycological well-being (theatre: 5.50 ± 0.52; visual: 4.90 ± 0.57; control: 4.97 ± 0.59, *p* = 0.001) (Noice et al., 2004). A significant improvement in cognitive parameters was also observed in a recent RCT by Cetinkaya et al. (2019) in a sample of 30 patients (15 in the intervention group and 15 in the control group) living in a nursing home. After an eight-week, twice-weekly ceramic painting session of approximately 30–35 min led by an art specialist, the participants in the intervention group showed significant improvement in Mini-Mental State Examination scores (24.2 ± 3.6) compared to the control group (21.4 ± 4.3) (*p* < 0.001) (Cetinkaya et al., 2019).

In the prospective 10-year study by Fancourt and Steptoe (2018), involving 3,445 participants with a mean age of 62.9 ± 7.5 years, the authors demonstrated that attendance at galleries and museums or the theatre—i.e., receptive activities—was associated with a smaller decline in cognitive function than non-attendance. More frequent attendance at such artistic activities improved memory and had a protective effect on semantic fluency, in contrast to going to the cinema, which showed a weak relationship and consequently a slight protective effect in the relationship with memory improvement and semantic fluency (Fancourt and Steptoe, 2018).

### 3.5. Art engagement and well-being

Seven studies, with different study design investigated the role of engagement with art in improving well-being (Table 6). In the RCT, conducted on a sample of 60 participants (intervention group: *n* = 30, control group: *n* = 30, mean age 72.6 ± 1.0), Aydin and Kutlu (2021) compared the effect of 6 weeks of weekly art therapy with clay (60–90 min) on loneliness and hopelessness with the effect of two 30-min sessions of face-to-face discussions on issues of health, social life and finances. At the end of 6 weeks, participants in the intervention group reported statistically significantly better scores on the UCLA Loneliness Scale (41.03 ± 10.33) and the Beck Hopelessness Scale (5.10 ± 2.32) than those who had participated in the face-to-face talk (UCLA-LS: 50.87 ± 10.94, BHS: 10.03 ± 2.50), although there was also significant improvement in the control group in the pre-post comparison. Similar results were also reported by Noice et al. (2004), who showed how physiological well-being significantly improved in participants who took part in dramatic and visual activities (drama: 5.50 ± 0.52, visual: 4.90 ± 0.57, control: 4.97 ± 0.59, *p* = 0.001) (Aydin and Kutlu, 2021).

**Table 6:** Results of studies analysing the impact of arts engagement on well-being and quality of life.

Author	Sample	Intervention sample	Comparator sample	Male/female ratio	Age	Endpoints	Outcome	Outcome measurement	Results
Aydin and Kutlu (2021)	60	30	30	13/47	72.6 ± 1.0	Baseline; After 6 weeks	Loneliness, Hopelessness	UCLA Loneliness Scale (UCLA-LS), Beck Hopelessness Scale (BHS)	UCLA-LS: statistically significant difference between IG and CG group after art therapy (IG: 41.03 ± 10.33, CG: 50.87 ± 10.94, *p* < 0.001) BHS: statistically significant difference between IG and CG group after art therapy (IG: 5.10 ± 2.32; CG: 10.03 ± 2.50, *p* < 0.001). Improvement in both group in pre-post comparison
Beauchet et al. (2020)	130	NA	NA	11/119	71.6 ± 4.9	Baseline (M0), after one months (M1), after second month (M2), and the third month (M3)	QoL and well-being	EuroQoL-5D (EQ-5D) - Warwick-Edinburgh Mental Well-being Scale (WEMWBS)	WEMWBS M0: 57.2 ± 7.4; M1: 57.3 ± 7.5; M2: 55.8 ± 9.1; M3: 57.5 ± 7.9 (M0 vs. M2: *p* = 0.040; M2 vs. M3: *p* = 0.004) EQ-5D: M0: 6.8 ± 2.0; M1: 6.4 ± 1.5; M2: 5.0 ± 1.1; M3: 4.8 ± 0.9 (M0 vs. M1: *p* = 0.004; M0 vs. M2: *p* ≤ 0.001; M0 vs. M3: *p* ≤ 0.001; M1 vs. M2: *p* ≤ 0.001; M1 vs. M3: *p* ≤ 0.001; M2 vs. M3: *p* ≤ 0.001)
Ho et al. (2019)	1067	1067	NA	479/588*missing values	50–59: 421 (39.5%); 60–69: 372 (34.9%); ≥70:: 274 (25.7%)	Cross-sectional	Quality of life; Physical and mental well-being; Spiritual well-being; Social well-being	WHO Quality of Life Instrument (WHOQoL-8); Short Form 20 (SF-20); Functional Assessment of Chronic Illness Therapy-Spiritual (FACIT-SP-12); Support Evaluation List Short Form (ISEL-S)	Passive engagement in arts and culture-related events experienced higher quality of life (*t*(728) = 3.35, *p* = 0.0008, *d* = 0.25), perceived health (*t*(728) = 2.21, *p* = 0.0277, *d* = 0.16) and sense of belonging (*t*(728) = 2.17, *p* = 0.03, *d* = 0.16), as compared with those who did not. Active engagement in participatory arts experienced greater quality of life (*t*(442) = 3.68, *p* = 0.0003, *d* = 0.36), self-rated health (*t*(442) = 2.59, *p* = 0.0099, *d* = 0.25), spiritual well-being (*t*(442) = 3.75, *p* = 0.0002, *d* = 0.37), meaning in life (*t*(442) = 5.03, *p* < 0.0001, *d* = 0.50) and sense of peace (*t*(442) = 3.72, *p* = 0.0002, *d* = 0.36), as compared with those who did not actively engaged in the arts.
Johnson et al. (2021)	69	Ceramics intervention: 17; Creative writing intervention: 12	31	Ceramics: 17/17; Creative writing intervention: 4/8; Control group: 4/27	Ceramics: 69.3 ± 7.9; Creative writing intervention: 66.1 ± 9.7; Control group: 73 ± 7.8	Baseline; After 8 weeks	Interest in life; Loneliness; Personal Growth; Mastery; General Belongingness; Neighbourhood Cohesion	Interest in life (National Institutes of Health Toolbox Apathy Scale), Personal growth and Perceived mastery (Psychosocial and Lifestyle Questionnaire), Loneliness (NIH Toolbox Loneliness Scale), General belongingness (General Belongingness Scale), Perceived neighbourhood cohesion (Self-perception of neighbourhood social cohesion)	Ceramics: statistically significant improvements in perceived mastery (adjusted difference 0.5, 95% CI: 0.2–0.7, *p* = 0.003) and interest in life (adjusted difference: 0.3 95% CI: 0.1–0.6, *p* = 0.007). No statistically significant improvement in general belongingness (adjusted difference: 0.2, 95% CI: 0.1 to −0.0, *p* = 0.11); loneliness (adjusted difference: 0.0, 95% CI: −0.2–0.2, *p* = 0.99); personal growth (0.0, 95% CI: −0.2–0.2, *p* = 0.72); neighbourhood cohesion (0.0, 95% CI: −0.5–0.4, *p* = 0.8). Writing: no statistically significant improvements on the well-being outcomes
Noice et al. (2004)	124	Theatre: 44; Visual: 36	31	27/97	73.7 ± 5.99	At baseline; After 4 weeks	Well-being	Self-esteem scale, Psychological Well-Being Scale	Self-esteem: Theatre: 3.64 ± 0.37; Visual: 3.37 ± 0.40, Control: 3.49 ± 0.36, *p* > 0.05 Psychological Well-being: Theatre: 5.50 ± 0.52; Visual: 4.90 ± 0.57; Control: 4.97 ± 0.59, *p* = 0.001
Thomson and Chatterjee (2016)	40	40	NA	11/29	65-85 years	Baseline; After the session	Physiological well-being	Positive Affect and Negative Affect Schedule (PANAS) and Subjective wellness ad happiness (Visual Analogue Scale)	Positive PANAS: Pre: 27.96 ± 9.84; Post: 31.51 ± 10.95, *p* < 0.001; Negative PANAS: Pre: 15.93 ± 5.89, Post: 13.37 ± 4.01, *p* < 0.001; Wellness VAS: Pre: 60.88 ± 23.49, Post: 66.27 ± 22.07, *p* < 0.005; Happiness VAS: Pre: 60.32 ± 24.69, Post: 68.85 ± 21.86, *p* < 0.001
Tymoszuk et al. (2020)	2767	2767	NA	1274/1493	62.3 ± 7.1	At enrolment (2004/2005); After 10 years (2014/2015)	Experienced well-being; Evaluative well-being; Eudaimonic well-being	Pleasure domain of the Control, Autonomy, Self-realisation, Pleasure scale (CASP-19); Dieners’s life satisfaction scale; Self-realisation domain of the CASP-19 and Control/Autonomy domain of a shortened version of the CASP-19	In the fully adjusted models, short-term engagement was not longitudinally associated with well-being, but repeated engagement with the theatre/concerts/opera and museums/galleries/exhibitions was associated with enhanced eudaimonic well-being, and sustained engagement with these activities was associated with greater experienced, evaluative, and eudaimonic well-being.

In a recent mixed-methods study conducted on a sample of 69 patients (ceramic arts intervention: *n* = 17, creative writing intervention: *n* = 12, control group without any form of activity: *n* = 31), Johnson et al. (2021) reported significant improvement in many parameters of well-being in participants who took part in ceramic activities. They showed that participants who took part in such activities showed greater improvement in perceived mastery (adjusted difference 0.5, 95% CI: 0.2 to 0.7, *p* = 0.003) and interest in life (adjusted difference: 0.3 95% CI: 0.1 to 0.6, *p* = 0.007), while no statistically significant improvement emerged for loneliness (adjusted difference: 0.0, 95% CI: −0.2 to 0.2, *p* = 0.99); personal growth (0.0, 95% CI, −0.2 to 0.2, *p* = 0.72); and neighbourhood cohesion (0.0, 95% CI, −0.5 to 0.4, *p* = 0.8), while the same significant trend was not observed for participants in the 12-week creative writing intervention (Johnson et al., 2021).

Beauchet et al. (2020), who engaged a sample of 130 community-dwelling older adults aged 65 and over in a 12-week weekly arts-based activity called “Thursday at the Museum,” showed significant improvement on the Warwirck-Edinburgh Mental Well-Being Scale after just 2 months (M0: 57.2 ± 7.4, M1: 57.3 ± 7.5, M2: 55.8 ± 9.1, M3: 57.5 ± 7.9; M0 vs. M2: *p* = 0.040, M2 vs. M3: *p* = 0.004) (Beauchet et al., 2020). Similar results were also found in the study by Thomson and Chatterjee (2016), in which subjective well-being and satisfaction, as measured by the visual analogue scale, were statistically significantly improved (wellness VAS: pre: 60.88 ± 23.49, post: 66.27 ± 22.07, *p* < 0.005; happiness VAS: pre: 60.32 ± 24.69, post: 68.85 ± 21.86, *p* < 0.001) after only one session in which participants handled and discussed museum objects such as archaeological artefacts (amulets, flint tools, ceramics); artworks (engraving plates, prints); geological specimens (fossils, rocks, minerals) and zoological specimens (horns, shells, teeth).

Ho et al. (2019), who conducted a cross-sectional study on a large sample of 1,067 residents of a community in Singapore, showed that both active (e.g., creative activity) and receptive (e.g., viewing or listening) engagement with art played a positive role in physical and mental as well as social and spiritual well-being. In fact, receptive participation in arts and cultural events significantly increased perceived health (*p* = 0.0277) and sense of belonging (*p* = 0.03) compared to those who did not actively engage in the arts, while active participation in participatory arts events improved self-rated health (*p* = 0.0099), spiritual well-being (*p* = 0.0002), meaning in life (*p* < 0.0001) and sense of peace (*p* = 0.0002) (Ho et al., 2019).

The prospective 10-year longitudinal study by Tymoszuk et al. (2020), conducted in the UK on a sample of 2,767 English people aged ≥50 years, showed that well-being, as measured by various subscales from the CASP-19, did not improve in the case of short-term engagement, but that repeated engagement in theatres/concerts/operas and museums/galleries/exhibitions was significantly associated with improved eudaemonic well-being, and that sustained engagement in these activities was associated with greater experienced evaluative and eudaemonic well-being (Tymoszuk et al., 2020).

### 3.6. Art engagement and quality of life

Five studies specifically examined a possible role of engagement with art in improving quality of life (Table 6). Specifically, Beauchet et al. (2020) reported a significant increase in quality of life after only 1 month of participation in the “Thursday at the Museum” project (EQ -5D: M0: 6.8 ± 2.0; M1: 6.4 ± 1.5; M2: 5.0 ± 1.1; M3: 4.8 ± 0.9; M0 vs. M1: *p* = 0.004; M0 vs. M2: *p* ≤ 0.001; M0 vs. M3: *p* ≤ 0.001; M1 vs. M2: *p* ≤ 0.001; M1 vs. M3: *p* ≤ 0.001; M2 vs. M3: *p* ≤ 0.001). A similar trend was also seen in the study by Cetinkaya et al. (2019) after 8 weeks of ceramic painting activity (based on the Life Satisfaction Scale, IG: 10.6 ± 3.0; CG: 9.1 ± 3.8; *p* = 0.115); and in the study by Ho et al. (2019), who showed higher quality of life in participants who engaged in both receptive (*p* = 0.0008) and active (*p* = 0.0003) art.

## 4. Discussion

According to the World Health Organisation, by 2050, the global population of older people will have more than doubled to 2.1 billion (World Health Organization, 2020). In a society where age is no longer a parameter in judging a person’s abilities, and people over 65 are considered active and able to live a life of activities and satisfactions like adults in their 40s or 50s, programmes and support that slow down the mental and physical ageing of the healthy population are seen as the new frontiers in medicine, as they can act as preventive and protective approaches that are capable of reducing the negative effects of advancing age and, thus, minimising the pressures that the rapid growth of the older population places on social security and health care systems (Nachu et al., 2023).

Arts engagement and related therapies consist an approach that supports individuals to express themselves, acquire coping skills, increase resilience, improve interpersonal skills, resolve conflicts and problems, reduce stress, manage behaviours and increase self-esteem and confidence (Shukla et al., 2022). This systematic review was designed to examine the effects of engagement with the arts on cognitive parameters, quality of life and well-being in healthy populations. With the exception of two papers, studies mainly published in the last 6 years were considered.

Our results demonstrate that cognitive decline in healthy people can be significantly slowed by a range of active and receptive artistic activities such as painting, visiting museums and galleries or going to the theatre or opera. Unlike activities such as going to the cinema or TV, receptive and active artistic activities are stimulating experiences that reduce cognitive decline while increasing well-being and quality of life. The mechanism of action for these positive outcomes of engagement with art can be attributed to three main reasons: (1) Engagement with art enables participants to experience complex and stimulating activities that improve neural structure and brain function, thus providing a protective effect against cognitive decline and neurological degeneration. (2) It reduces stress by lowering systolic blood pressure reactivity and increasing cortisol levels. (3) It provides a continuous source of stimulation for the brain in everyday life, thereby reducing the deterioration of cognitive function and improving a range of cognitive changes that counteract cognitive decline (Fancourt and Steptoe, 2018). When engagement with art is practised through active participation, the positive effects can be achieved in a short period of time (eight or 12 weeks), whereas when engagement with art is practised passively, the response process to such activities can take longer.

The same processes that reduce or at least slow down cognitive decline are also the basis for the positive effects of engagement with art on well-being and quality of life. As shown in some neuropsychological studies, pleasurable activities can trigger positive affect and increase arousal, which has a positive effect on dopamine levels in the brain (Ashby et al., 1999; Allerhand et al., 2014). The process of artistic activity on the well-being, quality of life, and cognitive decline is complex and still unclear. Participation in cultural activities, even in a receptive form, provides a mechanism to display and resolve emotions through a multisensory experience that stimulates creative processes, which, in a positive cycle, stimulates memory, releases emotions and increases activity levels (Beauchet et al., 2020). Art therapy delivered in art museums, for example, has been shown to promote social connectedness and physiological well-being, stimulating a wide range of emotions (Bennington et al., 2016). We hypothesise that this positive effect can be explained not only by exposing the brain to aesthetic stimuli, but also by participating in a group where such activities are commonly done. Sharing experiences and feelings has been considered as a kind of integral part of the process of engagement and is associated with the positive elements of evaluative, experiential and eudaemonic well-being (Bone et al., 2022).

The importance of group activity for arts engagement should be explored in depth. In the included studies, we found that active engagement in a single session, as demonstrated in the study by Thomson and Chatterjee (2016), can produce a significant and immediate increase in psychological well-being, whereas such an immediate response was not observed in receptive engagement (Tymoszuk et al., 2020), where only sustained engagement in specific arts activities (i.e., theatre, concert, opera, museums, galleries or exhibitions) was associated with a significant increase in experiential, evaluative and eudaemonic well-being.

Some questions were asked about the definition of *well-being* and *quality of life*. Although the two concepts are related and overlap accordingly, they could lead to different outcomes, especially when assessing the impact of receptive arts engagement for older adults, as well-being and quality of life in this specific population could be affected by health problems or negative experiences throughout life and may not have similar meaning to each individual (Pinto et al., 2017). Psychological well-being is a complex concept that includes hedonic and eudaemonic elements; it includes experienced well-being (which encompasses affective aspects such as positive and negative affect, i.e., feelings of happiness or depressed moods) and evaluative well-being (which concerns perceptions of quality of life, i.e., life satisfaction) (Tymoszuk et al., 2020).

From the evidence presented in this paper, it appears that the relationship between arts engagement and the outcomes considered here is well established. Nevertheless, some limitations have emerged. First, although our plan was to examine the role of the arts on some specific outcomes, the included studies focus only on the older population, as there is paucity of studies conducted on younger population that met our eligibility criteria. This means that the role of arts engagement in young and adult populations should be still established.

Secondly, the experimental studies did not take into account possible lifelong arts engagement of the participants: This means that the relationship between lifelong arts engagement and the prevention of cognitive decline and the improvement of well-being and quality of life is not established. Similarly, the role of interest in the arts aroused by the various activities proposed was not investigated, and no conclusions could be drawn about the long-term impact of the experimental activity.

Thirdly, the mediating role of group activities was not investigated in the included studies. This means that the positive effect of cultural engagement is not only a relevant factor related to the specific activity but could also be the result of social interaction, which, although not codified in this sense, could be a relevant component of the concept of cultural engagement: Interactions between individuals that lead to the sharing of information, experiences, views and common life problems could reduce the burden of negative feelings and increase the incentive to maintain a certain level of active lifestyle. Additionally, arts engagement may vary by race/ethnicity and socioeconomic factors, as well as by education level, parental education, income, social class and residence in a more urban area. With the exception of the longitudinal studies, the other included studies did not take such factors into account when evaluating responses. For example, education can increase engagement, raise awareness of activities and increase cognitive skills for engagement and influence engagement in the arts throughout the life course (Bone et al., 2021). This means that responses to arts engagement activities can vary widely across different socio-economic contexts.

Finally, qualitative studies were not included, as reported above in the method section. In future research, a sequential qualitative evidence synthesis that integrates our findings with evidence from interviews, focus groups, or case studies could help to deepen the role of arts engagement by examining how participants experience the arts and what the reasons are that bring people to the arts and increase their engagement.

## 5. Conclusion

In conclusion, the current evidence shows that in healthy older people, active and receptive arts engagement plays a fundamental role in slowing cognitive decline and ensuring high levels of well-being and quality of life. However, the role of such engagement in the adult population is unclear and the potential involvement of the young and adult population in more arts activities has not been explored in depth. The arts have been shown to be an important factor in social, community and personal enrichment, but their role in developing safeguards to reduce the burden of an ageing population has not been explored. Research in this area is essential to understand not only the positive impact of the arts on quality of life, well-being and cognitive decline but also on the mechanisms underlying these positive responses in order to develop programmes that can guide individuals through life and provide both a preventive strategy and non-pharmacological treatment approach for many diseases.

## Author contributions

MF conceived the study and carried it out together with MGR and MLG. MLG developed the search strategy and carried it out. MGR checked the references and the accuracy of the reported data. All authors contributed equally to the preparation of the manuscript.

## Conflict of interest

The authors declare that the research was conducted in the absence of any commercial or financial relationships that could be construed as a potential conflict of interest.

## Publisher’s note

All claims expressed in this article are solely those of the authors and do not necessarily represent those of their affiliated organizations, or those of the publisher, the editors and the reviewers. Any product that may be evaluated in this article, or claim that may be made by its manufacturer, is not guaranteed or endorsed by the publisher.

## Footnotes

^1^https://methods.cochrane.org/bias/resources/rob-2-revised-cochrane-risk-bias-tool-randomized-trials

^2^https://www.nhlbi.nih.gov/health-topics/study-quality-assessment-tools
